# Improving Delivery of Secondary Prophylaxis for Rheumatic Heart Disease in a High‐Burden Setting: Outcome of a Stepped‐Wedge, Community, Randomized Trial

**DOI:** 10.1161/JAHA.118.009308

**Published:** 2018-07-17

**Authors:** Anna P. Ralph, Jessica L. de Dassel, Adrienne Kirby, Clancy Read, Alison G. Mitchell, Graeme P. Maguire, Bart J. Currie, Ross S. Bailie, Vanessa Johnston, Jonathan R. Carapetis

**Affiliations:** ^1^ Menzies School of Health Research Darwin Northern Territory Australia; ^2^ Charles Darwin University Darwin Northern Territory Australia; ^3^ Royal Darwin Hospital Darwin Northern Territory Australia; ^4^ National Health and Medical Research Council Clinical Trials Centre University of Sydney New South Wales Australia; ^5^ Telethon Kids Institute University of Western Australia Perth Western Australia Australia; ^6^ Princess Margaret Hospital for Children Perth Western Australia Australia; ^7^ Baker Heart and Diabetes Institute Melbourne Victoria Australia; ^8^ University of Sydney University Centre for Rural Health Lismore New South Wales Australia; ^9^ Medical School Australian National University Australian Capital Territory Canberra Australia; ^10^ Perth Children's Hospital Perth Western Australia Australia

**Keywords:** acute rheumatic fever, adherence, cluster randomized trial, quality improvement, rheumatic heart disease, systems of care, Rheumatic Heart Disease, Secondary Prevention, Quality and Outcomes

## Abstract

**Background:**

Health system strengthening is needed to improve delivery of secondary prophylaxis against rheumatic heart disease.

**Methods and Results:**

We undertook a stepped‐wedge, randomized trial in northern Australia. Five pairs of Indigenous community clinics entered the study at 3‐month steps. Study phases comprised a 12 month baseline phase, 3 month transition phase, 12 month intensive phase and a 3‐ to 12‐month maintenance phase. Clinics received a multicomponent intervention supporting activities to improve penicillin delivery, aligned with the chronic care model, with continuous quality‐improvement feedback on adherence. The primary outcome was the proportion receiving ≥80% of scheduled penicillin injections. Secondary outcomes included “days at risk” of acute rheumatic fever recurrence related to late penicillin and acute rheumatic fever recurrence rates. Overall, 304 patients requiring prophylaxis were eligible. The proportion receiving ≥80% of scheduled injections during baseline was 141 of 304 (46%)—higher than anticipated. No effect attributable to the study was evident: in the intensive phase, 126 of 304 (41%) received ≥80% of scheduled injections (odds ratio compared with baseline: 0.78; 95% confidence interval, 0.54–1.11). There was modest improvement in the maintenance phase among high‐adhering patients (43% received ≥90% of injections versus 30% [baseline] and 28% [intensive], *P*<0.001). Also, the proportion of days at risk in the whole cohort decreased in the maintenance phase (0.28 versus 0.32 [baseline] and 0.34 [intensive], *P*=0.001). Acute rheumatic fever recurrence rates did not differ between study sites during the intensive phase and the whole jurisdiction (3.0 versus 3.5 recurrences per 100 patient‐years, *P*=0.65).

**Conclusions:**

This strategy did not improve adherence to rheumatic heart disease secondary prophylaxis within the study time frame. Longer term primary care strengthening strategies are needed.

**Clinical Trial Registration:**

URL: http://www.anzctr.org.au. Unique identifier: ACTRN12613000223730.

**Clinical Perspective**

**What Is New?**

- This multicomponent intervention at 10 remote Australian Aboriginal clinics did not improve adherence to penicillin among people with rheumatic fever or rheumatic heart disease during a 12‐month time frame, demonstrating the substantial challenges to effective adherence support.
- Adherence was already improving overall in this setting; the proportion receiving the nominated target of ≥80% of scheduled injections was 46% at baseline, more than double the previously published rate.
- Although improvement in the primary outcome was not seen, patients who were already well engaged experienced benefit during longer term follow‐up.
- Longer interventions are needed, with more community linkages, to achieve better adherence in this cross‐cultural context.

**What Are the Clinical Implications?**

- Secondary prophylaxis with penicillin is a highly effective strategy to reduce progression from acute rheumatic fever to rheumatic heart disease or from milder to more severe forms of rheumatic heart disease, but strategies to support adherence that are culturally appropriate and age appropriate are needed.
- The challenges identified in this study make a compelling case for investment in better preventive strategies at primordial and primary levels.
- More patient‐centered approaches within culturally competent health systems are a priority to achieve improved outcomes for individuals living with rheumatic heart disease.

## Introduction

Rheumatic heart disease (RHD), which develops as a complication of acute rheumatic fever (ARF), is an important cause of morbidity and mortality in areas of socioeconomic deprivation globally.^1^, ^2^ ARF is an abnormal immunological response that occurs in susceptible hosts after infection with group A streptococcus. In recognition of the high morbidity and mortality burden caused by RHD, the World Health Assembly adopted a resolution on RHD in May 2018 acknowledging the need for a comprehensive approach to the prevention and control of the disease in endemic countries. In the Indigenous population living in Australia's Northern Territory (NT), very high rates are observed: Among 5‐ to 14‐year‐old Indigenous children, the annual incidence of ARF is 250 per 100 000^3^ and prevalence of RHD is up to 1500 per 100 000.^4^ RHD accounts for one of the greatest differential disease rates between Indigenous and non‐Indigenous Australians,^2^ with Indigenous people 55 times more likely to die from ARF or RHD.^5^ Cumulative cardiac valvular damage from recurrent ARF leads to RHD. The overall incidence of progression to RHD has been estimated recently in our setting at 51.9% within 10 years after initial ARF.^6^

The most effective strategy to prevent ARF recurrences and RHD progression is secondary prevention with intramuscular long‐acting benzathine penicillin G (BPG) injections every 4 weeks for a minimum 10 years after the last ARF episode or until age 21, whichever occurs later.^7^ Continued prophylaxis to age 35 is recommended for moderate RHD and to age 40 or lifelong for severe RHD, especially in those requiring valve surgery.^7^ Challenges to delivery of this regimen in remote Australian settings are similar to those in many resource‐poor populations globally with the highest RHD burdens: high turnover and limited ARF knowledge among healthcare staff; young and mobile patients; pain of injections; and cultural factors leading to different concepts of disease causation and treatment, along with barriers to acceptance of Western medicine.^8^, ^9^, ^10^ The proportion of patients in the NT in 2009 with ARF/RHD achieving ≥80% of scheduled injections—the current Australian benchmark for acceptable adherence^7^—was only ≈25%.^11^

We hypothesized that barriers to adherence would be amenable to a health system intervention, based on elements of the chronic care model (CCM)^12^ and using continuous quality improvement (CQI) processes. The CCM for chronic disease management incorporates 6 domains: health systems, delivery system design, decision support, clinical information systems, self‐management support, and community linkages.^12^ These domains have high applicability to ARF/RHD management. Interventions based on the CCM have the potential to improve health outcomes.^13^, ^14^ CQI approaches use data proactively to motivate healthcare providers and clinic managers to work toward improved targets. Application of CQI has shown promise previously in the NT, with implementation at 6 remote clinics leading to improvements in some ARF/RHD outcomes.^11^ Health systems improvements providing a “comprehensive care program” or combining education strategies with development of a register have also been associated with ARF rate reductions in the United States, Cuba, and the Caribbean.^15^, ^16^, ^17^ Nevertheless, methodological aspects of previous studies may limit the inferences that can be drawn and extended to other settings.

We aimed to test whether a health systems intervention engaging staff and patients at primary care clinics and the regional RHD control program would improve delivery of secondary prophylaxis for people with ARF and/or RHD. A comprehensive mixed‐methods evaluation done in parallel is reported separately.^18^

## Methods

### Study Design

The RHDSP (Rheumatic Heart Disease Secondary Prophylaxis) trial was a stepped‐wedge, pragmatic, community, randomized trial with an open cohort design using mixed‐methods evaluation. The study protocol was published previously.^19^ A stepped‐wedge trial is an alternative to a parallel cluster trial design. After an initial period of no intervention, clusters (in this case, pairs of communities) commence the intervention at regular intervals (steps). Data are collected throughout, allowing for the staggered intervention phases to be compared with the baseline phases. The study was conducted in Australia's NT, in Indigenous communities, most of them in very remote locations. Each has a single primary healthcare center (clinic) servicing the community. Patients can be mobile between communities but have a “primary clinic” nominated (the clinic takes responsibility for patients’ treatment, where the majority of their injections are delivered).

Approval was provided by the Human Research Ethics Committee of the NT Department of Health and Menzies School of Health Research (no. 2012‐1756) and the Central Australian Human Research Ethics Committee (no. 2013‐126). The trial was registered with the Australian New Zealand Clinical Trials Registry: ACTRN 12613000223730. The anonymized quantitative data and study materials will be been made available on request to the authors for purposes of reproducing the results or replicating the procedure.

### Participants

The unit of participation was healthcare centers, which were eligible if they had ≥10 patients with ARF and/or RHD requiring secondary prophylaxis and provided written informed consent from the health center manager or management board. Of note, in Australia, the term *Indigenous* includes Aboriginal and Torres Strait Islander peoples; in our study, all Indigenous people were Aboriginal Australians, reflecting the demographics of the Indigenous NT population. Consent from individuals was not required for quantitative data collection; these data were routinely collected and reported to the regional RHD Control Program. For qualitative data collection, healthcare providers at participating clinics, patients with ARF and/or RHD, and key stakeholders involved in ARF care were invited to participate in interviews. Informed consent for interviews was sought by project officers or a qualitative researcher using written and verbal materials, with information provided in an Australian Indigenous language, if appropriate. For patients aged <15 years, consent was sought from a parent or guardian and assent was requested from the interviewee.

### Randomization and Masking

Clusters in the stepped‐wedge study comprised pairs of health centers that entered at 3‐month steps in random order. The random allocation code was computer generated centrally at the National Health and Medical Research Council Clinical Trials Center. No components of the intervention were subject to masking.

### Procedures

**Figure 1:**
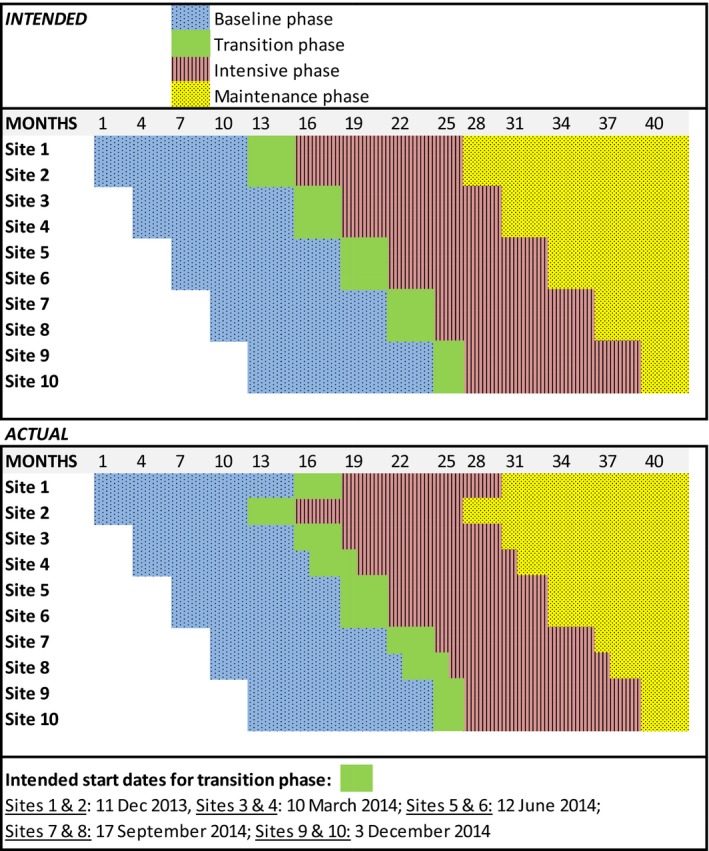
Stepped‐wedge study design.

Study phases comprised (1) baseline data collection (12 months), (2) transition phase with commencement of intensive phase activities but data excluded from outcome analyses (3 months); (3) intensive implementation phase (12 months), (4) maintenance implementation phase (3–15 months, depending on each site's start date; Figure 1).^19^

The intervention comprised a set of activities (“action plans”) aligned under the themes of the CCM and developed and implemented by health centers. Implementation was supported by project officers via monthly face‐to‐face meetings during the transition and intensive phase (ie, 15 times) and every 3 months during the maintenance phase. CQI activities comprised meetings between the staff of participating heath centers and project officers to review quarterly adherence rates, presented as simple graphics, and discussion of progress against action plans. If clinics were unable to be visited because of closure, such as for a funeral or flooding, project officers sought a meeting by telephone. All efforts were made to ensure that exposure to the intervention, measured as the number of clinic support visits conducted face‐to‐face or by telephone, was homogeneous across sites and in accordance with the stepped‐wedge design.

### Outcomes

The a priori primary outcome was proportion of patients receiving ≥80% of their scheduled BPG injections over the 12‐month intensive phase compared with the baseline phase. Secondary outcome measures were proportion of scheduled injections that a patient received; average number of “days at risk” of ARF due to late penicillin dosing (median number >28 days between scheduled injections)^20^ in a 12‐month period; proportion of patients in other adherence strata (90–100%, 50–79% and <50% of scheduled BPG injections); recurrence rate and proportion of ARF episodes that were recurrences compared with nonparticipating communities and the whole jurisdiction; impact of the intervention on RHD patients’ experience of care, including their perception and understanding of the disease and its management; improvement in delivery of other services for RHD patients; and effect of the program on delivery of other routine services.

### Data

Adherence data (penicillin injection dosing with date and location of administration) were obtained from the NT RHD Control Program Register. A subset of these data was checked for accuracy against primary clinic data, and corrected if required, for the whole study period, as reported elsewhere.^21^ The NT Aboriginal Health Key Performance Indicator (AHKPI) data—an aggregated, deidentified data set—were used to measure the secondary outcome of impact of the intervention on other services. Prespecified indicators selected were AHKPI 1.4.1: Fully Immunized Children; AHKPI 1.7: Chronic Disease Management Plan for diabetic patients; AHKPI 1.11: Adult Health Check, aged ≥55 years; AHKPI 1.12: Pap Smear Tests in the previous 2 years.

“One21Seventy” RHD audits^18^, ^21^ were used to measure aspects of ARF/RHD care other than penicillin adherence, such as whether a timely echocardiogram and cardiologist review had been undertaken. These audits are an initiative of the National Center for Quality Improvement in Indigenous Primary Health Care and provide deidentified data. Community and clinic characteristics were derived from the Australian Bureau of Statistics and project officer reports. Staff stability was defined as the average number of months that a single person was in the RHD nurse coordinator role at each healthcare center during the transition and intensive phases; a higher score indicates greater staffing stability.

Qualitative data collection comprised semistructured interviews with ARF/RHD patients, clinic staff, and key stakeholders and project officer reports summarizing each episode of contact with a healthcare center. A nested focused ethnographic study (description of cultural behavior focused on ARF and RHD) was conducted at 4 sites, investigating patients’ perspectives on their condition.

### Project Evaluation

A program theory was developed using the 6 themes of the CCM as the framework.^19^ We hypothesized that a cascade of potential outcomes arising from these activities would occur, leading to increased adherence and thus reduction in ARF recurrence rates. Effectiveness of implementation of the project was measured according to fidelity, dose, and reach. Fidelity included whether participating healthcare centers developed action plans and implemented activities as planned; dose comprised the number and nature of scheduled contacts achieved between clinics and project officers (face‐to‐face visits as intended or conducted by phone); reach of the intervention comprised the number and quality of action items and comprehensiveness across the CCM themes. Evaluation findings are reported separately.^18^

### Statistical Analyses

Using a stepped‐wedge design with a 3‐month period for each step, with enough communities to provide at least 30 participants at each step and taking into account within‐cluster correlation, 300 patients were required to provide 90% power and a 2‐sided significance level of 5% to detect an increase from 20% to 40% of participants receiving ≥80% of their scheduled penicillin injections.

The intervention was implemented at the level of the community health center, but measured by its impact on ARF/RHD patients and health center staff. All patients with ARF and/or RHD whose primary clinic was participating in the study were included. In an a priori plan taking account of population mobility, analyses were restricted to those who, during the baseline and intensive phases of the study, resided in a study site for at least 75% of the time (≥9 of 12 months during each phase of quantitative data collection) and required penicillin prophylaxis for ≥12 months during each of the baseline and intensive phases of the study. To avoid potential adherence rates of >100% and counting multiple needles administered within a short time frame, adherence calculations excluded doses given within 14 days of each other; however, all doses were retained in calculations of days at risk.

Analyses of adherence were based on observations for each participant in each time period and used generalized linear mixed models to account for the correlation within a person over time and between people within the same clinic. The primary analysis included only the baseline and intensive phases. When the maintenance phase was included in the analysis, the results showed the maintenance phase compared with the baseline and intensive phases, with a test for trend over the 3 time points. Binary outcomes included whether a patient received ≥80%, ≥90%, <50%, and 50% to 79% of scheduled BPG injections over the 12‐month intensive phase. Days at risk of ARF recurrence were presented in 2 ways: number of days >28 days (or >21 for those prescribed 21‐day regimens) that occurred between scheduled injections in a 12‐month period or proportion (days at risk divided by number of days that penicillin was intended). Days at risk were Poisson distributed, and proportion of days at risk was normally distributed and analyzed using a generalized linear mixed model. Results are presented as odds ratios, rate ratios, or absolute differences and 95% confidence intervals. Patients had to be included in both baseline and intensive phases to be included in the analysis. Comparison of ARF recurrence rates was undertaken using MedCalc software. All other analyses were undertaken in SAS 9.3 (SAS Institute).

The impacts of the study on delivery of other services for RHD patients and on other routine clinic activities were assessed using One21Seventy and AHKPI data, respectively. For several reasons, graphical descriptions rather than statistical tests were undertaken for these outcome measures: the time frames for aggregated data sets did not exactly align with the stepped‐wedge design, not all sites undertook One21Seventy audits, and One21Seventy data are deidentified so could not be used in individual patient‐level adherence analyses.

Healthcare provider interview transcripts and project officer reports were subjected to deductive coding by 3 researchers in NVivo qualitative data analysis software (v10, 2012; QSR International Pty Ltd). Codes were determined a priori according to established criteria. Interpretation of findings was made in consultation with the project staff and other study investigators. Patient interview transcripts were analyzed using an exploratory inductive approach.

### Role of the Funding Source

The trial was funded by the Australian National Health and Medical Research Council and Wesfarmers Center for Vaccines and Infectious Diseases at Telethon Kids Institute. The funders had no role in the study design, data collection, analysis, interpretation, or writing of this report.

## Results

**Figure 2:**
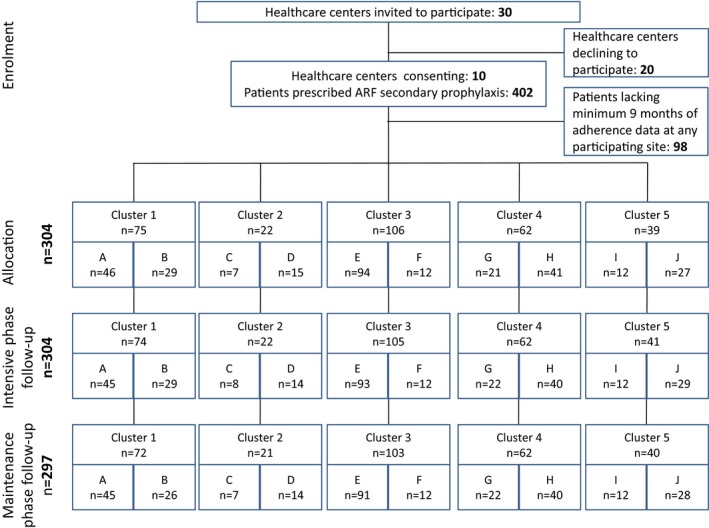
CONSORT diagram. Sample sizes show the number of people in the cluster receiving prophylaxis. Some patients moved between study sites and some left the study (died, no longer required prophylaxis, moved to a nonparticipating site), accounting for the slight differences in numbers shown at each site during different study phases. ARF indicates acute rheumatic fever.

We approached 30 health centers about participation. Ten agreed to participate, providing 402 participants at study commencement. Factors associated with nonparticipation included a sense of “research fatigue” and concern about added work burden the study might generate. The Template for Intervention Description and Replication checklist^23^ is provided in Table S1. Ninety‐eight participants were excluded for having <9 of 12 months of available adherence data at any participating study site during the intensive phase (Figure 2), leaving 304 for analysis. Some patients moved between study sites, and some left the study (died, no longer required prophylaxis, moved to a nonparticipating site), accounting for slight differences in numbers available at each site during different study phases. Key study dates are shown in Figure 1. Health center characteristics are shown in Table 1. Chief attributes were that most communities were classified as *very remote* (eg, in 1 instance, >7‐hour drive on unsealed roads from the nearest regional center), with small populations but high numbers of individuals per household and low incomes. Four clinics were run by community‐controlled health boards and 6 by the Government Department of Health. Staff turnover was high: During the 15‐month period of data collection from start of transition to end of intensive phase, the number of healthcare providers at each study site holding the portfolio of RHD coordinator ranged from 2 to 8 (Figure S1). Only 1 site provided an outreach (community‐based) service.

**Table 1:** Baseline Community, Healthcare Center, and Patient Characteristics

Characteristics	A	B	C	D	E	F	G	H	I	J
Communities										
Community population^a^	1171	3062	842	1100	2292	685	6094	14 600	454	1528
Remoteness^b^	Very remote	Very remote	Very remote	Very remote	Very remote	Very remote	Remote	Outer regional	Very remote	Remote
Average no. people per household^a^	6.4	4.5	4.7	6	5.3	5.4	3.3	3.2	6.1	4.7
Average no. people per bedroom^a^	2.4	1.8	2.2	2.3	2.2	1.9	1.6	1.3	2.1	1.7
Median weekly household income, AUD^a^	$1346	$904	$1954	$1270	$1920	$1140	$1367	$1378	$719	$724
Healthcare centers										
Governance of clinic	Government	Community	Community	Community	Government	Government	Community	Community	Community	Government
Patients^c^	46	29	7	15	94	12	21	41	12	27
Female, n (%)	16 (35)	12 (41)	2 (29)	10 (67)	31 (33)	3 (25)	9 (43)	13 (32)	3 (25)	8 (30)
Age, y, median (IQR)	25 (18–32)	23 (19–30)	19 (15–24)	25 (11–35)	24 (16–31)	25 (15–33)	29 (16–38)	27 (14–38)	26 (18–31)	28 (17–40)
≤10	1 (2)	0 (0)	0 (0)	2 (13)	3 (3)	1 (8)	2 (10)	4 (10)	1 (8)	4 (15)
10.1–20	15 (33)	12 (41)	4 (57)	5 (33)	34 (36)	4 (33)	5 (24)	10 (24)	2 (17)	5 (19)
20.1–40	25 (54)	16 (55)	3 (43)	8 (53)	46 (49)	7 (58)	10 (48)	20 (49)	8 (67)	13 (48)
40.1–60	5 (11)	1 (3)	0 (0)	0 (0)	11 (12)	0 (0)	4 (19)	7 (17)	1 (8)	5 (19)
Receiving ≥80% of scheduled injections, n (%)	17 (37)	2 (7)	3 (43)	10 (67)	59 (63)	4 (33)	7 (33)	12 (29)	3 (25)	24 (89)
Days at risk, median (IQR)	135 (56–209)	197 (147–243)	102 (65–149)	60 (41–105)	73 (46–122)	93 (58–140)	137 (83–219)	155 (69–256)	132 (98–178)	53 (32–64)

AUD indicates Australian dollars; IQR, interquartile range.

a 2011 Australian census.

b Australian Standard Geographical Classification—remoteness areas.

c Patients were eligible if in the community and prescribed penicillin for acute rheumatic fever for a minimum 9 months in the intensive and baseline phases.

### Adherence Outcomes

Primary and secondary outcomes are presented in Table 2. The proportion of patients receiving ≥80% of scheduled BPG injections was 141 of 304 (46%) in the baseline phase and 126 of 304 (41%) during the intensive phase (odds ratio: 0.78; 95% confidence interval [CI], 0.54–1.11]). During the maintenance phase, this proportion increased to 148 of 297 (50%; odds ratio compared with baseline: 1.18 [95% CI, 0.81–1.72]; odds ratio compared with intensive phase: 1.55 [95% CI, 1.07–2.26]; Table 2). The proportion of patients receiving ≥90% of scheduled BPG injections was 92 of 304 (30%) during the baseline phase. An increase occurred in this adherence measure in the maintenance phase to 128 of 297 (43%), compared with the intensive phase (odds ratio: 2.43; 95% CI, 1.63–3.62). The number of days at risk (Table 3) was not significantly different during the intensive phase (median: 98 days; interquartile range: 56–177) compared with baseline (median: 86 days; interquartile range: 49–162; rate ratio: 0.95 [95% CI, 0.89–1.02]). However, the proportion of days at risk decreased in the maintenance phase (0.28 versus 0.32 [baseline] and 0.34 [intensive], *P*=0.001). ARF recurrence rates did not differ between study sites during the intensive phase and the whole jurisdiction (3.0 versus 3.5 recurrences per 100 patient‐years, *P*=0.65). There was no interaction between the prespecified subgroups (age, sex, site) and change in adherence.

**Table 2:** Outcomes Showing the Effect of the Study Intervention on Measures of Adherence in the Intensive Phase and Subsequent Effect in the Maintenance Period

				Adherence Comparison 2 Phases Primary Analysis		Adherence Comparison for All 3 Phases		
				Intensive vs Baseline Phase		Maintenance vs Baseline^a^	Maintenance vs Intensive^a^	
Adherence Measure	Baseline Phase, n (%)	Intensive Phase, n (%)	Maintenance Phase, n (%)^a^	OR (95% CI)	*P* Value	OR (95% CI)	OR (95% CI)	*P* Value for Trend^b^
Primary outcome						Secondary analyses		
≥80%^c^	141/304 (46)	126/304 (41)	148/297 (50)	0.78 (0.54–1.11)	0.16	1.18 (0.81–1.72)	1.55 (1.07–2.26)	0.38
Secondary outcomes								
90%–100%^c^	92/304 (30)	85/304 (28)	128/297 (43)	0.87 (0.59–1.28)	0.47	2.09 (1.41–3.09)	2.43 (1.63–3.62)	<0.001
50%–79%^c^	98/304 (32)	112/304 (37)	93/297 (31)	1.25 (0.88–1.77)	0.21	0.95 (0.67–1.37)	0.76 (0.54–1.09)	0.81
<50%	65/304 (21)	66/304 (22)	56/297 (19)	1.03 (0.67–1.60)	0.88	0.84 (0.54–1.31)	0.82 (0.53–1.27)	0.45
				Mean difference (95% CI)				
Percentage received, mean (SD)	70 (25)	69 (27)		−0.5 (−2.7 to 1.7)	0.67			
				Rate ratio (95% CI)		Mean difference (95% CI)	Mean difference (95% CI)	*P* value for trend
Average no. days at risk per 12‐mo period, median (IQR)^d^	86 (49–162)	98 (56–177)	e	1.05 (0.98–1.13)	0.17			
				Mean difference (95% CI)				
Proportion of days at risk, mean (SD)^a^	0.32 (0.02)	0.34 (0.02)	0.28 (0.02)	0.02 (0.001–0.05)	0.04	−0.04 (−0.06 to −0.02)	−0.06 (−0.09 to −0.04)	0.001

CI indicates confidence interval; IQR, interquartile range; OR, odds ratio.

a Proportion of days at risk is days at risk divided by number of penicillin injections intended.

b Trend across the 3 time periods (baseline, intensive, maintenance).

c Percentage of scheduled benzathine penicillin G injections over a 12‐mo period, with analyses adjusted for clinic.

d Days at risk is the number of days >28 days (or >21 where appropriate) that occurred between scheduled injections.

e Not tested because maintenance phase was <12 mo at the majority of sites.

**Table 3:** Intervention Delivery and Adherence Outcomes at the 10 Participating Sites

Intervention Delivery	Community									
	A	B	C	D	E	F	G	H	I	J
Commenced at intended time	No—3 mo late	Yes	Yes	No—1 mo late	Yes	Yes	Yes	No—1 mo late	Yes	Yes
No. months of maintenance phase follow‐up	12	15	12	11	9	9	6	5	3	3
Face‐to‐face meetings, n^a^	9	12	14	12	13	11	12	13	7	12
Telephone meetings, n^b^	0	3	0	2	0	0	1	0	2	0
Total visits, n (%)	9/15 (60)	15/15 (100)	14/15 (93)	14/15 (93)	13/15 (87)	11/15 (73)	13/15 (87)	13/15 (87)	9/15 (60)	12/15 (80)
Action items^c^	1	13	12	10	4	3	3	10	5	4
Staff turnover index^d^	1.88	7.5	7.5	7.5	3	3	7.5	7.5	5	3.75
Undertook One21Seventy audit in baseline phase	Yes	Yes	Yes	Yes	Yes	Yes	Yes	Yes	No	Yes
Undertook One21Seventy audit in intensive phase	No	Yes	No	Yes	No	Yes	Yes	No	No	Yes
Provided AHKPI data	Yes	Yes	Yes	Yes	Yes	Yes	Yes	Yes	No	Yes

AHKPI indicates Aboriginal Health Key Performance Indicator.

a Number of face‐to‐face support visits from project staff at allocated time.

b Number of telephone support sessions substituting for noncompleted face‐to‐face meetings.

c Number of completed core action items by end of intensive phase.

d The average number of months that a single person was in the rheumatic heart disease nurse coordinator role at each healthcare center during the 15‐mo transition and intensive phase (ie, higher numbers indicate greater staffing stability).

**Figure 3:**
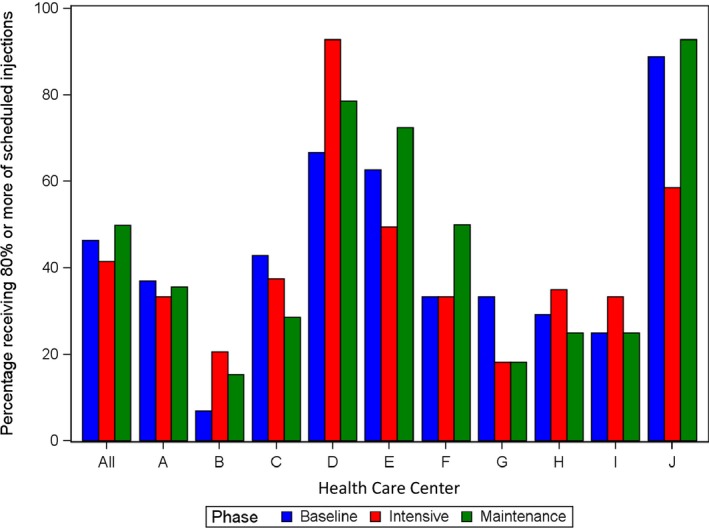
Proportion of patients receiving ≥80% of scheduled benzathine penicillin doses during baseline, intensive, and maintenance phases at all sites and according to individual study site.

There was a large difference between sites in adherence (Table 4 and Figure 3): the proportion of patients receiving ≥80% of injections at baseline ranged from 7% to 89% (*P*<0.001). Differences in adherence also occurred between age groups (Table 4), being best in children ≤10 years and worst in the group aged 21 to 40 years (*P*<0.001). Adherence did not differ between male and female patients.

**Table 4:** Proportion of Patients Who Received ≥80% of Their Scheduled Injections by Prespecified Subgroups, Adjusted for Site During 24 Months (Baseline and Intensive)

Subgroup	Category	≥80% During Baseline Phase, n (%)	≥80% During Intensive Phase, n (%)	Total Adherence, n (%)	Intensive vs Baseline–OR (95% CI)	Between‐Groups Comparison–OR (95% CI)	*P* Value for Difference Between Groups	*P* Value for Interaction in Primary Outcome Analysis
Age group	≤10 y	13/18 (72)	10/18 (56)	23 (64)	0.37 (0.06–2.06)	1.05 (0.42–2.62)	<0.001	0.19
	11–20 y	43/96 (45)	49/96 (51)	92 (48)	1.34 (0.72–2.50)	0.64 (0.35–1.18)		
	21–40 y	63/156 (40)	49/156 (31)	112 (36)	0.60 (0.36–1.02)	0.36 (0.20–0.64)		
	41–60 y^a^	22/34 (65)	18/34 (53)	40 (59)	0.45 (0.12–1.64)	1		
Sex	Female^a^	48/107 (45)	44/107 (41)	92 (43)	0.82 (0.45–1.50)	1	0.48	0.89
	Male	93/197 (47)	82/197 (42)	175 (44)	0.77 (0.50–1.19)	0.88 (0.61–1.27)		
Site	A	17/46 (37)	15/45 (33)	32 (35)	···^b^	0.20 (0.09–0.41)	<0.001	0.13
	B	2/29 (7)	6/29 (21)	8 (14)	···	0.06 (0.02–0.15)		
	C	3/7 (43)	3/8 (38)	6 (40)	···	0.24 (0.07–0.80)		
	D	10/15 (67)	13/14 (93)	23 (79)	···	1.39 (0.47–4.10)		
	E	59/94 (63)	46/93 (49)	105 (56)	···	0.47 (0.24–0.90)		
	F	4/12 (33)	4/12 (33)	8 (33)	···	0.18 (0.06–0.51)		
	G	7/21 (33)	4/22 (18)	11 (26)	···	0.12 (0.05–0.31)		
	H	12/41 (29)	14/40 (35)	26 (32)	···	0.17 (0.08–0.36)		
	I	3/12 (25)	4/12 (33)	7 (29)	···	0.15 (0.05–0.43)		
	J^a^	24/27 (89)	17/29 (59)	41 (73)	···	1		

a Reference category.

b Numbers per site too small for comparison.

### ARF Recurrences

Recurrence rates of definite or probable ARF diagnosed using the Jones criteria during intensive phase were compared between the 10 participating sites and the whole NT during the relevant period (March 1, 2014, to February 29, 2016). During the intensive phase, 9 definite or probable ARF recurrences occurred at participating sites in individuals identified as needing secondary prophylaxis (3.0 recurrences per 100 patient‐years), compared with 3.5 recurrences per 100 patient‐years in the whole jurisdiction (*P*=0.66).

### Determinants of Adherence and of Change in Adherence

No associations were seen between adherence and objectively quantifiable characteristics of health centers, communities, or project activities including patient numbers per site, staff turnover, participation or not in One21Seventy audits, socioeconomic indicators, numbers of action items completed, number of study visits achieved, or governance structure (Table 4, Figure S2). Multivariable analyses using clinic‐level characteristics as predictors of response could not be undertaken because outcomes were analyzed on a per‐patient basis, and analysis on a per‐clinic level would have been inappropriate because of having only 10 outcome variable data points but many clinic‐level predictor variables, potential correlation between the predictor variables, and no replication of measures. These data are described in Tables 1 and 2. However, there were specific case examples illustrating the complex interplay of factors that might influence adherence. One site achieving high adherence during the intensive phase (93% of patients getting ≥80% of scheduled injections; Figure 3, siteD) had community‐controlled governance, stable staffing, a small ARF patient caseload (<30 individuals), very remote location, One21Seventy audits, 13 of 15 scheduled visits completed, and implementation of 10 action items during the intensive phase. In contrast, a site with low adherence during the intensive phase (33% of getting ≥80%; Figure 3, site I) had higher staff turnover, undertook only 5 action items, had 9 of 15 study visits, and did not participate in One21Seventy audits, yet, like site D, also had community‐controlled governance, a small caseload, and very remote location. Such factors are explored in detail in the project evaluation.^18^

### Project Implementation

Regarding fidelity to the protocol, all participating sites successfully developed action plans to address penicillin delivery for ARF/RHD patients (Table S1); however, 3 sites experienced delays (Figure 1 and Table 2). Examples of action plan items are provided in Table S2—for example, changing electronic patient recall systems to ensure early reminders of penicillin doses and triaging ARF/RHD patients to high priority to avoid long waiting times for injection delivery. The dose of the intervention ranged from 7 of 15 face‐to‐face visits at one site up to 14 of 15 at another (Table 2). The reach of the intervention varied with numbers of completed action items ranging from 1 to 13 (Table 2, Figure S2Q). Project officer report data indicated that activities continued into the maintenance phase.

### Impact of the Intervention on RHD Patients’ Experience of Care

The focused ethnographic study exploring patients’ experiences of care at sites A, C, D, and G involved 35 ARF/RHD patients (aged 7–35 years) and 37 family members or Aboriginal key informants. Main themes are presented in summary in this article; detailed qualitative findings are presented elsewhere.^18^ A key theme that emerged was that patient knowledge of ARF/RHD and the reason for regular injections was very limited. Patients had many unanswered questions about their conditions that they felt powerless to ask. This was compounded by communication difficulties given the need to operate in a second language (English) in health services. Patients generally did not recount a discernible impact from the intervention. Some did note value in being able to access injections outside the clinic via outreach services and receiving reminder text messages via mobile telephone from clinics. Others perceived that the only change over time affecting their care was staff turnover.

### Impact of the Study on Other Clinic Activities

NT AHKPI data from the participating sites are shown in Figure S3. Adherence to ARF treatment according to the AHKPI data set showed close association with our study data set, indicating validity of the AHKPI data (Figure S3E). The proportions of individuals receiving guideline‐compliant care in the nominated categories (immunization, chronic disease management, adult health check, Pap smear) showed no overall association with the study's primary outcome and no suggestion of an impact of the study on the other clinic activities. Clinic performance in other indicators generally exceeded performance in ARF prophylaxis, and interclinic variability in performance was greatest for ARF prophylaxis. RHD file audit results (One21Seventy audits) were examined to look for associations between penicillin adherence and other aspects of ARF/RHD care. Results at the 5 participating sites that undertook audits during baseline and intensive phases are shown in Figure S4. The figure suggests that sites achieving the best adherence results also did well in ensuring that people had timely echocardiography, dental review, and influenza vaccination. However, associations with adherence were not apparent at all sites (eg, site B), and no association was evident between response to the study intervention and performance in other aspects of ARF/RHD care.

## Discussion

In this first rigorous randomized controlled trial to test the impact of a health systems intervention on delivery of RHD secondary prophylaxis, no significant increase in adherence was achieved. Improvement in documented adherence was already evident at the outset of this study: double (46%) the proportion of patients were receiving ≥80% of scheduled injections compared with the previous estimate (23%).^24^ The intervention was unable to achieve a further incremental improvement over this background rise. Twelve months may have been insufficient to detect a true effect, given the complex intervention we implemented. There was improvement during the maintenance phase in the proportion of patients receiving ≥90% of scheduled injections. This suggests, first, that patients already engaging well gained the most from the intervention, whereas those who were struggling did not benefit—they need to be reached by other means. Second, it suggests that the type of intervention implemented, which used CQI processes, may take longer to have effect than we anticipated when devising the primary outcome measure. This is supported by findings from a study using CQI to improve the delivery of diabetes mellitus care in Australian Indigenous communities: Longer duration of participation in CQI audit cycles, up to 6 years, was an important determinant of improved outcomes.^25^

Reasons for the overall increase in documented adherence in the NT may include the now‐widespread local practice (days at risk approach) of recalling patients for their 28‐day injection from day 21 onward to avoid late delivery.^20^ This practice has been gradually implemented since 2014 and was recommended at all our participating sites to avoid days at risk between penicillin doses. In addition, there have been ongoing efforts to strengthen primary health care throughout the NT, including CQI programs, for several years before this project commenced, and that may have contributed to the trends in improvement before baseline—this may have limited our ability to show an impact of our intervention. Furthermore, a spurious increase in adherence because of higher quality data may have occurred. Indeed, the data cleaning we undertook resulted in an 8‐percentage‐point increase in calculated adherence.^21^

Age and site were significant predictors of adherence. Reassuringly, adherence was best in children, who have the highest risk of ARF recurrence. Given methodological constraints—having only 10 participating sites for comparison, but with diverse characteristics—we were unable to identify individual community, clinic, or project implementation factors in statistical analyses that significantly predicted adherence or response to the study intervention. It appeared that a complex interplay of factors at high‐performing sites contributed to their ability to deliver care more effectively. Chief among these was the presence of stable staffing provided by culturally competent individuals working within well‐functioning clinics. Previous research in our setting has identified that staff who are able to identify with patients and healthcare centers that value quality improvement initiatives and are successful in developing effective community linkages respond more effectively to CQI initiatives.^26^ Objectively measuring the “culture” of a clinic, healthcare provider quality and health service cultural competence, is difficult—although research is under way to measure institutional cultural competence more effectively.^27^ Clinic governance differed between sites, with some under local Aboriginal community control and others being government‐run. Governance structure was not found to be associated with outcomes in this study (Table 4, Figure S2N).

A key factor that we believe impeded project success was staffing challenges, including high turnover (Table 4)—a well‐recognized problem in our setting^28^, ^29^ Solutions to rural health workforce shortages and turnover have been identified, including increased training and workplace support for Aboriginal people; strategies to develop professional development opportunities, peer support, and community connectedness for nonlocal staff; and placement of students to promote rural and remote career choices.^30^ Findings from our study suggest that greater effort is needed in implementing such strategies. At one site (A), the RHD portfolio was overseen by 8 different nurses during a 15‐month period. Although we sought to implement measures that would be durable in the face of staff turnover, project officers noted failures of handover, and patients noted the difficulty of engaging with a clinic where staff were very frequently new and unfamiliar. Despite these context‐specific findings, similar factors may operate internationally in settings with high RHD burdens. ARF and RHD occur at high rates among Indigenous populations of Canada, New Zealand, South America, and globally in resource‐limited settings^1^; many of our findings could be generalizable to such settings.

We used the CCM to guide and categorize the activities that clinics undertook to improve delivery of penicillin injections. Clinics found some aspects of chronic disease management easier to implement than others—the majority of implemented action items involved changing clinic‐based systems such as software‐embedded recall systems or undertaking staff training. Far more challenging was the ability to establish effective community linkages or to provide self‐management support. Among the 10 clinics, only 4 action items in the self‐management support domain were undertaken (Table S2). Self‐management has many definitions but is understood to be an approach to chronic disease management that acknowledges patients as active participants in their treatment and encourages them to make informed decisions about care and engage in healthy behaviors.^31^, ^32^ Self‐management is poorly named for our setting because Australian Aboriginal culture universally values group identity. A term such as “community group‐based care” would be more appropriate. Major investment is now required to conceptualize, develop, and implement effective “self‐management” strategies in our setting.

The AHKPI data attest to the added challenges of the delivery of RHD prophylaxis compared with other routine services, which were more likely to meet or approach targets (Figure S4). The RHD medical record audit data (One21Seventy audits) indicated some associations—high‐performing clinics did well across most aspects of ARF/RHD care—but the project activities seeking to improve adherence did not have apparent spill‐over effects, neither improving nor detracting from a focus in other domains.

A chief limitation of the study is the short time frame. Stepped‐wedge designs have advantages over traditional cluster randomized community trials,^18^ and this model was appropriate to use for this study. A disadvantage, however, is the added time requirement: We collected data for >3.5 years, but this provided only a 12‐month intensive intervention period. As per the stepped design, maintenance‐phase data were available for only 3 months from some sites, so results from this phase need to be interpreted with caution. There was heterogeneity of the intervention at different participating sites; however, we ensured it was as comparable and structured as possible while also allowing site‐specific tailoring to address differing clinic needs and to allow a sense of ownership of the project by the healthcare providers implementing the activities. Accounting for patient movements between sites was statistically complex; patients were not necessarily living at study sites for the whole period. We analyzed patient data according to their nominated primary clinic, but injections were also delivered at other sites (primary clinics supposedly take responsibility for notifying traveling patients and destination clinics that a penicillin dose is due). In addition, to reduce the impact of patient movements, we restricted analyses to patients who were at a study site for at least 75% of the intensive phase. Female patients were underrepresented in this study; they comprise ≈62% of individuals on the NT RHD register but only 35% in our study. Because adherence did not differ between male and female patients in our study, this is unlikely to have affected the study findings.

## Conclusion

Secondary prevention is the cornerstone of international ARF and RHD control, but effectiveness is limited by suboptimal adherence. Our findings indicate the critical importance of improving engagement between healthcare services and Aboriginal patients. The barriers to this improvement need to be addressed by engaging the highest levels of health system governance, using innovative staff education and retention measures and ensuring cultural security within healthcare environments. A wide range of other creative strategies need to be explored, such as use of social media, incentives, peer‐support groups, and consideration of community support personnel employed to assist Aboriginal people in navigating the healthcare system. The ongoing challenges in delivery of secondary prophylaxis make a compelling case to broaden the scope of ARF prevention activities, with particular emphasis on primordial and primary prevention both among individuals with existing ARF or RHD and among whole communities. Research now under way is engaging communities to address high streptococcal transmission in households and communities to provide community‐led solutions to reducing the strikingly high rates of RHD.

## Sources of Funding

This study was funded by the Australian National Health and Medical Research Council (NHMRC) project grant 1027040 and Center of Research Excellence 1080401, and by the Wesfarmers Center for Vaccines and Infectious Diseases at Telethon Kids Institute. Ralph and Maguire are supported by NHMRC fellowships (1142011 and 1046563 respectively).

## Disclosures

None.

## Supporting information


**Table S1.** TIDieR (Template for Intervention Description and Replication) Checklist
**Table S2.** Examples of Action Plan Items
**Figure S1.** Rheumatic heart disease coordinator staff turnover during the 15‐mo period of data collection from start of transition to end of intensive phase of the study.
**Figure S2.** Association between adherence (percentage of patients receiving ≥80% of scheduled injections during intensive phase) and clinic characteristics. No associations were found to be statistically significant at *P*<0.05.
**Figure S3.** Associations between Aboriginal Health Key Performance Indicators between 2013 and 2016, and adherence as calculated for this study during the intensive phase of the study.
**Figure S4.** Results of rheumatic heart disease audit results (“One21Seventy” audits of medical files) in relation to adherence to secondary prophylaxis.
