# Supplementary material for: Improving Delivery of Secondary Prophylaxis for Rheumatic Heart Disease in a High‐Burden Setting: Outcome of a Stepped‐Wedge, Community, Randomized Trial
Source: J Am Heart Assoc. 2018 Jul 17;7(14):e009308. doi: 10.1161/JAHA.118.009308 (PMC6064833; doi:10.1161/JAHA.118.009308)
Supplement: Supplementary file 1 — Table S1. TIDieR (Template for Intervention Description and Replication) Checklist Table S2. Examples of Action Plan Items Figure S1. Rheumatic heart disease coordinator staff turnover during the 15‐mo period of data collection from start of transition to end of intensive phase of the study. Figure S2. Association between adherence (percentage of patients receiving ≥80% of scheduled injections during intensive phase) and clinic characteristics. No associations were found to be statistically significant at P<0.05. Figure S3. Associations between Aboriginal Health Key Performance Indicators between 2013 and 2016, and adherence as calculated for this study during the intensive phase of the study. Figure S4. Results of rheumatic heart disease audit results (“One21Seventy” audits of medical files) in relation to adherence to secondary prophylaxis. [file JAH3-7-e009308-s001.pdf]

SUPPLEMENTAL MATERIAL

Table S1: TIDieR (Template for Intervention Description and Replication) checklist[1]

Item	Description
Name	A health systems intervention to improve delivery of secondary prophylaxis for people with acute rheumatic fever (ARF) and rheumatic heart disease (RHD).
Why	Adherence to secondary prophylaxis (SP) is sub-optimal. There are lost opportunities for prevention of ARF recurrences, which drive the progression to RHD. RHD has high morbidity and mortality. Factors important in the delivery of secondary prophylaxis include robust patient recall systems and care of patients which is culturally appropriate and engaging. An intervention targeted at health centres to improve these processes could increase adherence, and thereby, reduce ARF recurrences and progression to RHD.
What	***Documents**:* Partnership Agreement between clinic management (health centre manager or management board) and study investigator; Participant information forms and consent sheets provided for interviews.<br><br>***Materials**:* Printed reports and verbal discussion of health centre progress for continuous quality improvement cycles, based on 3-monthly summaries of their centre's adherence data, and progress with Action Plan items; study newsletter highlighting ideas and progress at all participatign sites; clinician and patient educational resources e.g. www.rhdaustralia.org.au/resources, www.rhdaustralia.org.au/health-worker-modules, www.rhdaustralia.org.au/clinician-modules<br><br>***Procedures***: collaborative development of Action Plan by healthcare providers and project team, to identify activities to improve SP delivery under the 6 themes of the Chronic Care Model. See Supplementary Table 3.<br>Interviews: interviews were undertaken to ascertain healthcare provider and client views on delivery and receipt of penicillin secondary prophylaxis, and to seek opinions from key stakeholders on health system structures to support ARF care.
Who provided	Health centre staff (doctors, nurses, Aboriginal Health Practitioners, managers) supported by two project officers (registered nurses) supporting five health centres each.<br>Logistical support provided by Project Manager and Project Coordinator (administration staff).<br>Clinical and other support and overall project supervision provided by study investigators (clinician researchers).
How	Face to face meetings: project officers visited communities regularly.<br>Support between visits: telephone and email.<br>Quarterly study newsletter distributed by email and provided in hard copy during site visits.
Where	Health centres at ten communities in the Northern Territory of Australia. These were widely spaced geographically - up to 2000 km apart.
When and how much	***Baseline phase***: 12 months of data collection without project officer visits<br>***Transition (wash in) phase:*** 3 months during which intensive phase activities were commenced but quantiative data collectino was not undertaken<br>***Intensive phase:*** 12 months of data collection with monthly scheduled face-to-face project officer visits<br>***Maintenance phase:*** 3-12 months of data collection with monthly telephone contact and 3-monthly face-to-face project officer visits
Tailoring	Each participating health centre was invited to create their own 'Action plan' to improve delivery of rheumatic fever prophylaxis. Project officers provided ideas to include in the Action Plan, ensured that actions were appropriate, determined that actions could be categorized under a theme of the Chronic Care Model and provided motivation and facilitation to complete action items.
Modifications	If face-to-face visits were unable to be achieved, these were replaced by telephone 'meetings'.<br>If clinics were unable to commence the study on the date allocated by the randomization schedule, all milestones for that site were adjusted accordingly.
How well	***Planned***: intervention adherence and fidelity were captured in regular project officer reports. Strategies to maintain fidelity included weekly staff meetings and quarterly chief investigator meetings to manage issues arising, regular checking of project progress against the protocol, and negotiation with clinic management when issues such as lack of site access arose.<br>***Actual:*** 'Dose' of intervention delivery (number of face-to-face visits) differed between sites as shown in the manuscript. Reasons for missed site visits included: health centre requested no visit due to competing priorities; health centre inaccessible due to flooding; health centre not operating routinely due to large community funeral or other event.<br>Degree of engagement with healthcare providers and health centre management varied between sites.<br>The number, quality and relevance of core action items completed by clinics varied between sites.

1. Hoffmann TC, Glasziou PP, Boutron I, Milne R, Perera R, Moher D, Altman DG, Barbour V, Macdonald H, Johnston M, Lamb SE, Dixon-Woods M, McCulloch P, Wyatt JC, Chan AW and Michie S. Better reporting of interventions: template for intervention description and replication (TIDieR) checklist and guide. *BMJ*. 2014;348:g1687.

Table S2. Examples of action plan items.

Chronic Care Model theme	Examples of activities undertaken at participating healthcare centres	Number of completed core action items by end of intensive phase										
		A	B	C	D	E	F	G	H	I	J	TOTAL
Clinical information systems	- Establish a list of all ARF secondary prophylaxis (SP) patients in the electronic medical record<br>- Set up an alert in the electronic medical record to flag SP patients<br>- Regularly cross-check and update the clinic list against the Control Program register list<br>- Change patient recall from 28 to 21 days to ensure patient has time to attend for needle by day 28<br>- Determine where patients prefer to receive their injection (e.g. clinic, home, school) and document this in a consistent place in the electronic medical record<br>- Develop regular data sharing process between adjacent communities for patients who live for period of time in both communities<br>- Develop a system for handing the RHD Portfolio over to future RHD coordinators	1	6	8	6	0	2	0	3	1	2	29
Community linkages	- Consult with the local school about the feasibility of injection administration at the school<br>- Liaise with community elders about a strategy for delivering injections during ceremonial business<br>- Provide information on ARF/RHD to a Women's community group ('Women's Yarning Circle')<br>- Incorporate RHD health promotion into Heart Week promotional activities	0	0	0	1	0	1	0	1	1	0	4
Decision Support	- Ensure all relevant staff have undertaken the RHD educational online modules e.g. include these in the staff orientation package and provide time for existing staff to do these<br>- Invite the NT RHD Control Program staff to visit the community to provide education to staff on RHD care<br>- Include a discussion of SP data as a standing agenda item for clinical staff meetings<br>- Ensure that all staff are familiar with pain minimisation techniques for administering intramuscular injections	0	3	1	2	2	0	3	2	2	1	16
Delivery System Design	- Ensure a nurse is assigned to the RHD Coordinator position and clarify the position description<br>- Ensure that triage and other processes for SP patients are fast tracked<br>- Build a new cubicle in the waiting room to fast track injection delivery<br>- Ensure all SP patients have a current penicillin prescription<br>- Identify strategies for administering injections to children during the school e.g. determine where children will be spending the school holidays and liaise with the relevant community clinic<br>- Implement the use of vibrating ice packs ('Buzzy Bee™') for pain minimisation; document in the electronic medical record who wishes to have this device used<br>- Allocate an RHD program day which is not Monday or Friday to avoid clinic closures and public holidays	0	3	1	1	1	0	0	2	1	1	10
Health Systems	- Discuss RHD at the clinic board meeting and explain the significance of the disease and the importance of SP. Get input from board members on ways to strengthen delivery of care.<br>- Clarify the role of the NT RHD Control Program team (what they can do to support the clinic)	0	1	1	0	0	0	0	0	0	0	2
Self Management Support	- Encourage staff to undertake online Self Management Support training	0	0	1	0	1	0	0	2	0	0	4

	- Introduce ways to remind patients of when their injection is due: reminder cards/ phone calls/ text message / calendar in phone / wall calendar<br>- Develop a smartphone application (app) to provide patients with reminders about injections; Educate patients about the app<br>- Design hand-held reminder cards in appropriate Aboriginal language<br>- Distribute wall calendars to patients<br>- Distribute adherence certificates to patients<br>- Obtain feedback from patients on which reminder method they prefer											

ARF: acute rheumatic fever; injection: refers to injections of benzathine penicillin G; NT: Northern Territory; SP: secondary prophylaxis; RHD: rheumatic heart disease

Figure S1. RHD coordinator staff turnover during the 15-month period of data collection from start of transition to end of intensive phase of the study.

Month	A	B		C	D	E		F	G	H		I	J
1	1	1	2	1	1	1		1	1	1	2	1	1
2	2	1	2	1	1	1		2	1	1	2	1	1
3	3	1	2	1	1	2		2	1	1	2	1	1
4	2	1	2	1	1	2		2	1	1	2	1	1
5	2	1	2	1	1	2		2	1	1	3	1	1
6	2	1	2	1	1	3		***	2	1	4	1	2
7	2	1	2	1	1	3		2,3	2	1	4	1	2
8	4	1	2	2	1	3		3	2	1	4	***	2
9	2	1	2	2	1	4	5	3	2	1	4	***	3
10	2	1	2	2	1	4	5	3	2	1	4	2	3
11	5	1	***	2	1	4	5	3	2	1	4	2	3
12	5	1	***	2	1	4	5	3	2	1	4	2	3
13	6	1	***	2	1	4	5	4	2	1	4	2	***
14	7	3	***	2	1	4	5	5	2	1	2	2	4
15	8	3	***	1	2	6	***	***	2	1	2	2	4
Total:	8	3		2	2	6		5	2	4		2	4

*** : no RHD coordinator

At each of the 10 participating sites, the nurse (or in one instance, an Aboriginal Health Practitioner) assigned the RHD Coordinator role is signified by a number and corresponding colour. At some sites during all or part of the 15-month period, two individuals were assigned the role. In some sites an individual left and then later returned to the role.

Figure S2. Association between adherence (percentage of patients receiving ≥80% of scheduled injections during intensive phase) and clinic characteristics. No associations were found to be statistically significant at $p<0.05$

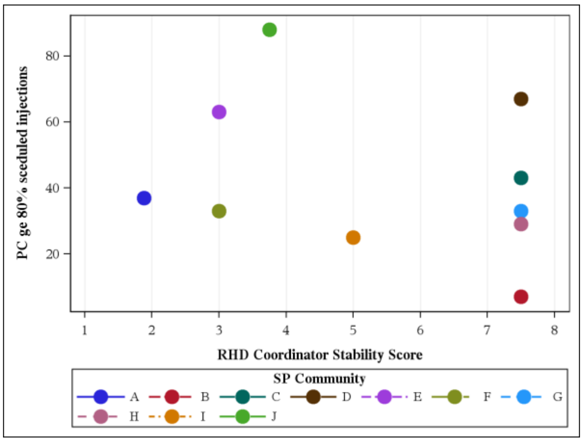

A. Adherence by RHD nurse coordinator turnover stability score. Higher value indicates higher turnover.

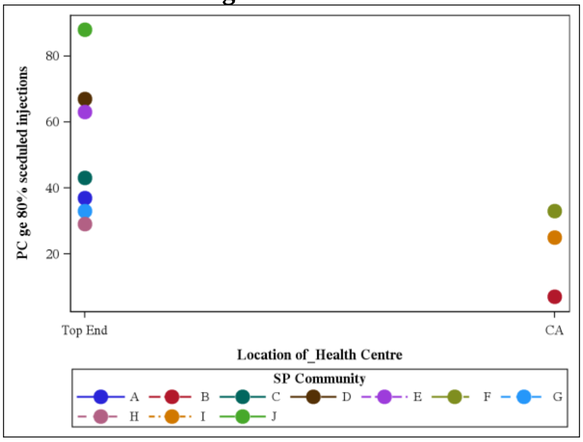

B. Adherence by geographical location of clinic in the Top End or Central area of the Northern Territory

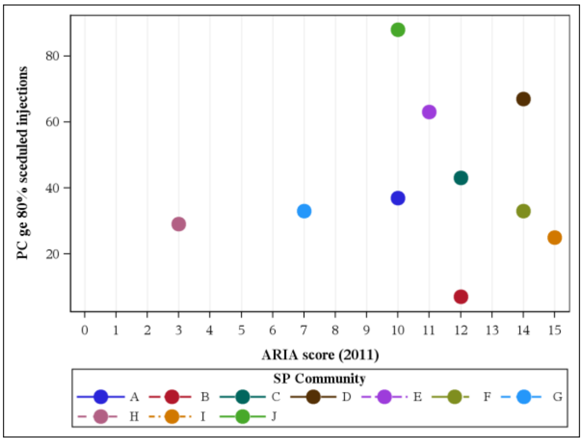

C. Adherence by Accessibility/remoteness index of Australia (ARIA) score

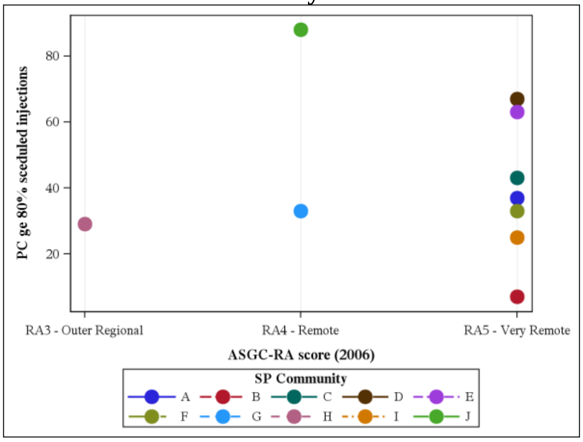

D. Adherence by Australian statistical geography standard-remoteness area score

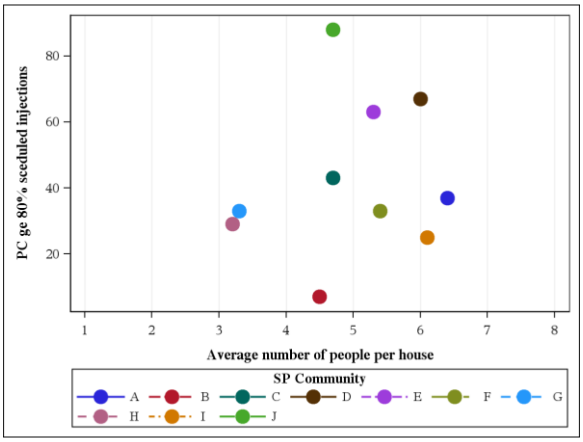

E. Adherence by household crowding: number of people per house

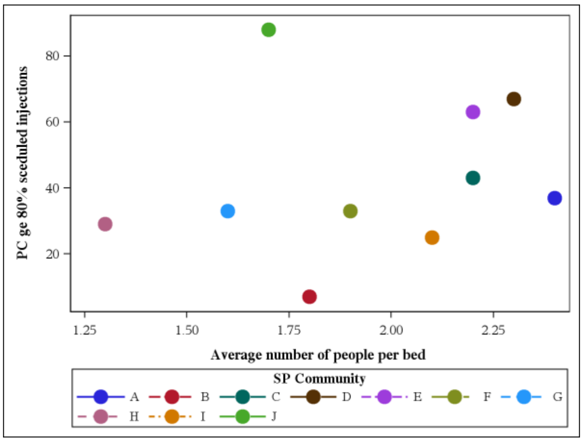

F. Adherence by household crowding: number of people per bed

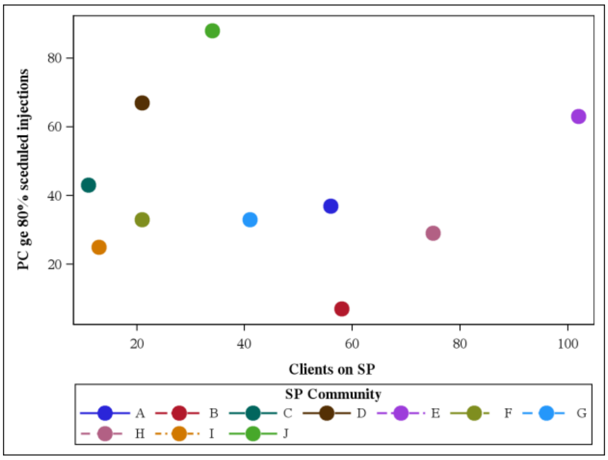

G. Adherence by number of patients in the community needing ARF prophylaxis

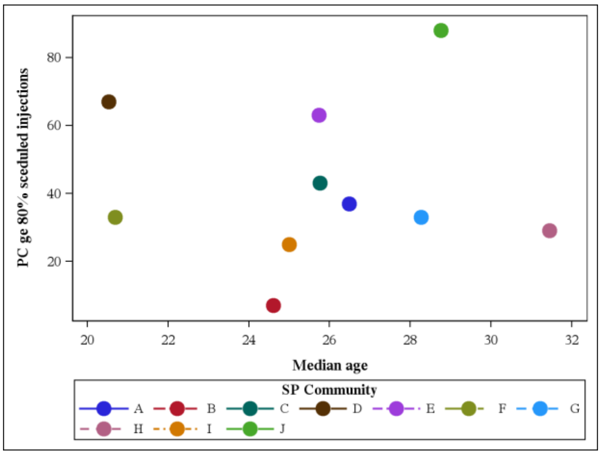

H. **Adherence by age of patients in the community needing ARF prophylaxis**

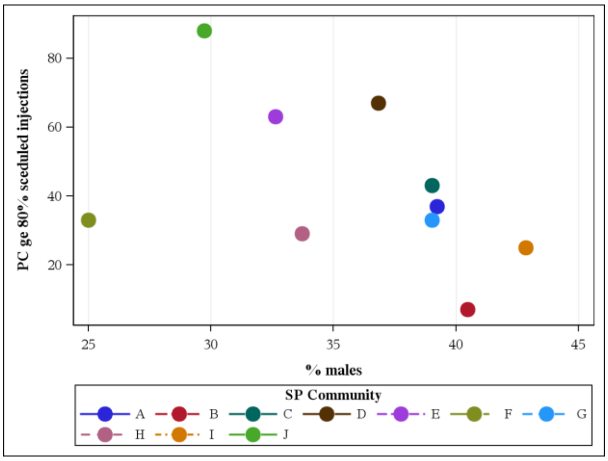

I. Adherence by proportion of patients in the community needing ARF prophylaxis who are male

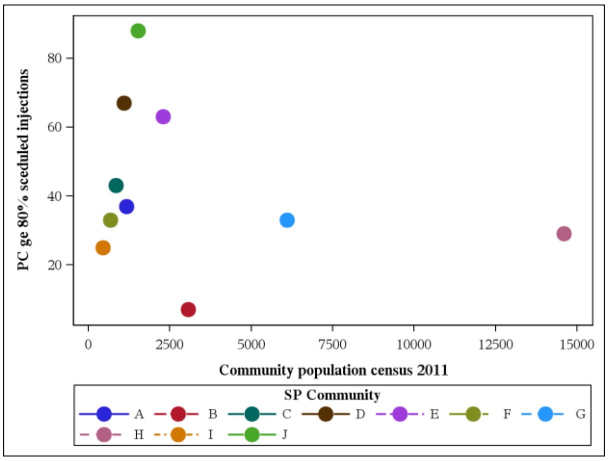

J. Adherence by community population

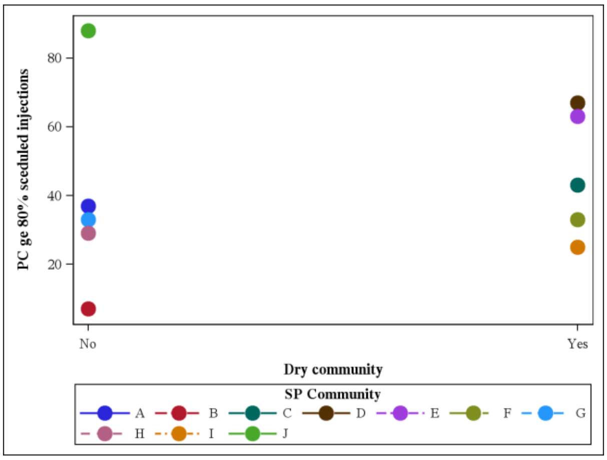

K. Adherence by availability of alcohol in the community (dry = alcohol not legally obtainable)

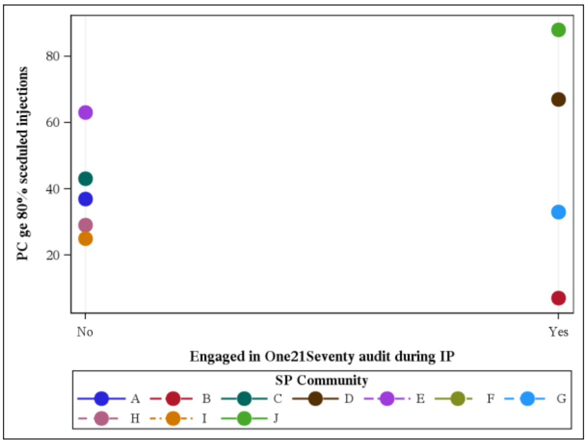

L. Adherence by participation of the clinic in 'One21Seventy' audits in the intensive phase

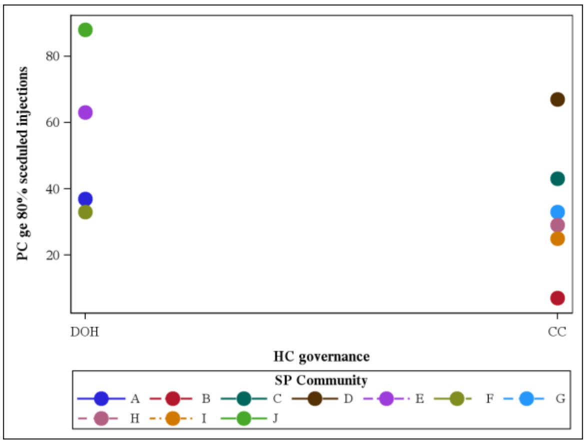

N. Adherence by type of governance of clinics (Government Department of Health (DOH) or Community Controlled (CC))

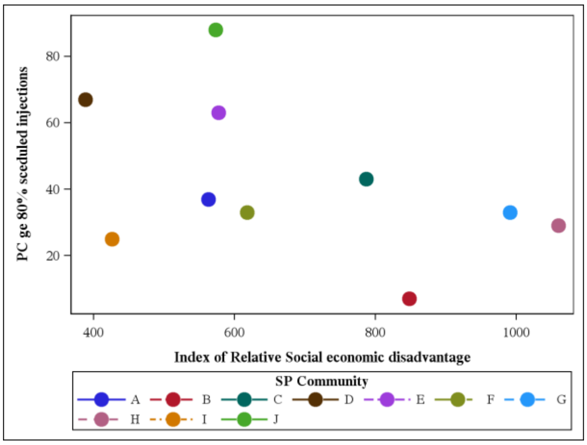

O. Adherence by index of relative social economic disadvantage (lower score means less advantaged)

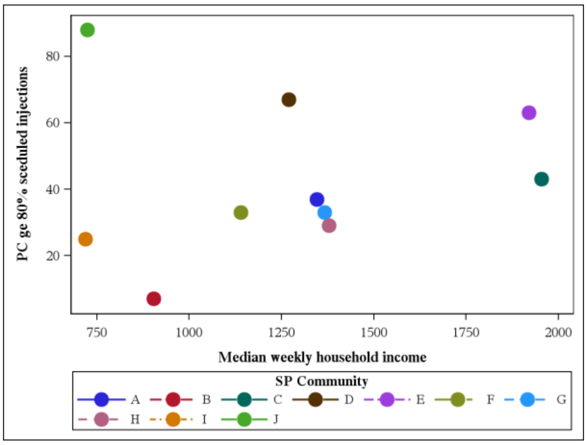

P. Adherence by median weekly household income

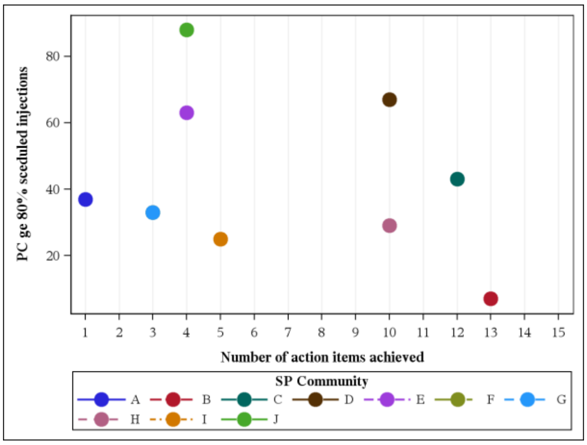

Q. Adherence by number of action items acheived

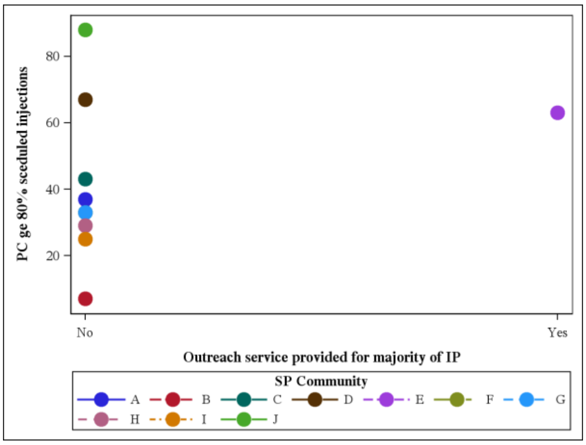

R. Adherence by provision of outreach (community-based) services for patients requiring ARF prophylaxis during intensive phase

Figure S3. Associations between Aboriginal Health Key Performance Indicators between 2013 and 2016, and adherence as calculated for this study during the intensive phase of the study.

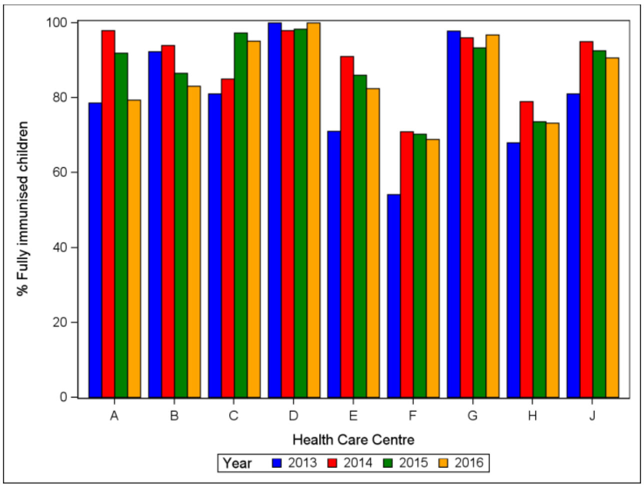

A. Proportion of children fully immunised

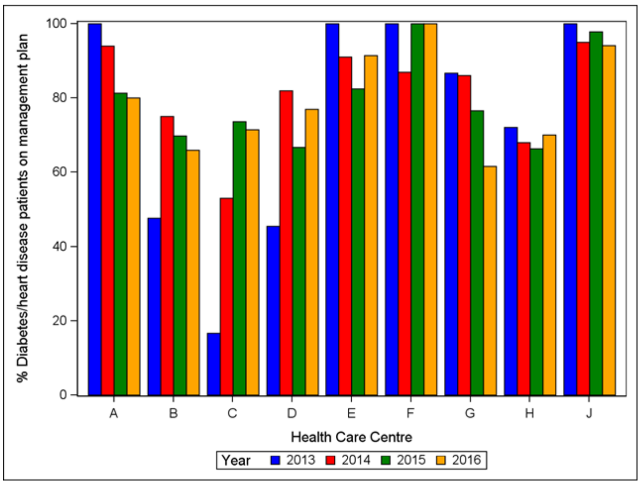

B. Proportion of people with diabetes and chronic heart disease with a management plan in place

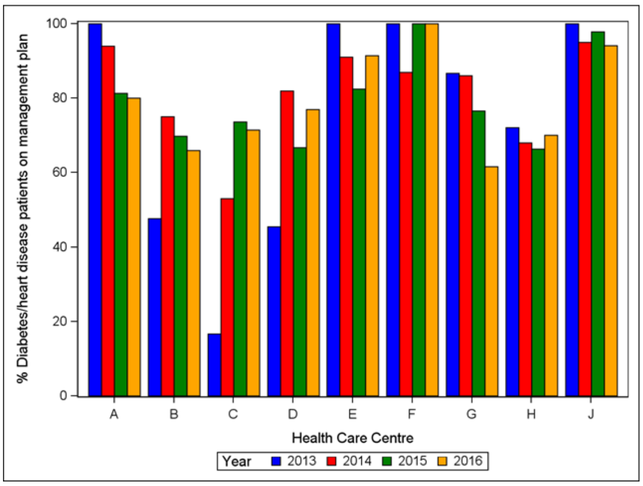

C. Proportion of adults aged >55 years receiving an annual health check

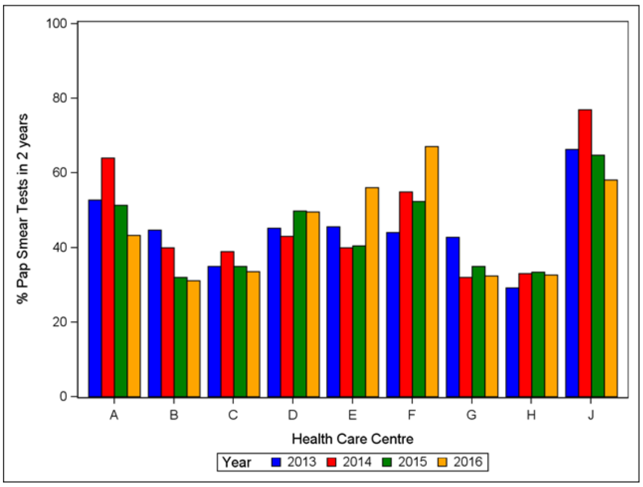

D. Proportion of women in required age range having a pap smear every two years

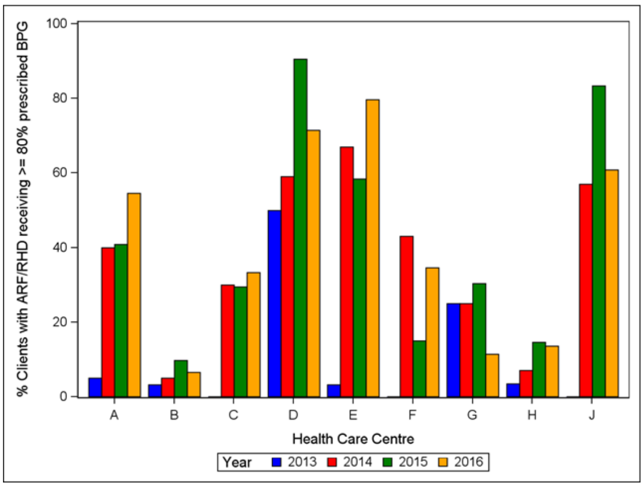

E. Proportion of patients with ARF receiving ≥80% of scheduled BPG injections as documented in the AHKPI

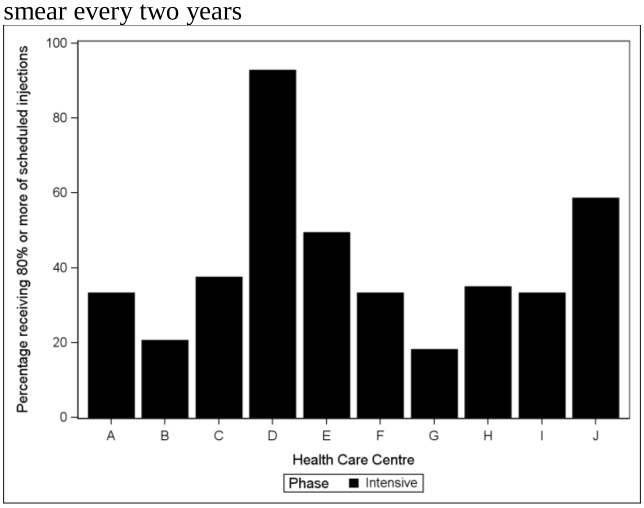

F. Adherence at each site during the intensive phase of the study, as a comparison with AHKPIs shown in A-E

Colored bars show the percentage of all patients at the clinic who had the variable of interest documented. Note, access to AHKPI data was not provided by Site I. Black bars shows the primary outcome i.e. proportion of patients receiving ≥80% of scheduled BPG injections during the intensive phase. The intensive phase occurred at different time intervals depending on the random order in which sites entered the study.

Figure S4. Results of rheumatic heart disease audit results (‘One21Seventy’ audits of medical files) in relation to adherence to secondary prophylaxis.

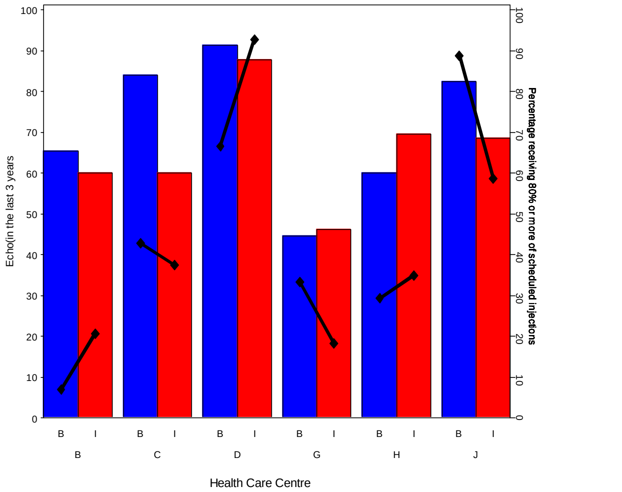

A. Documentation of echocardiogram in the last 3 years

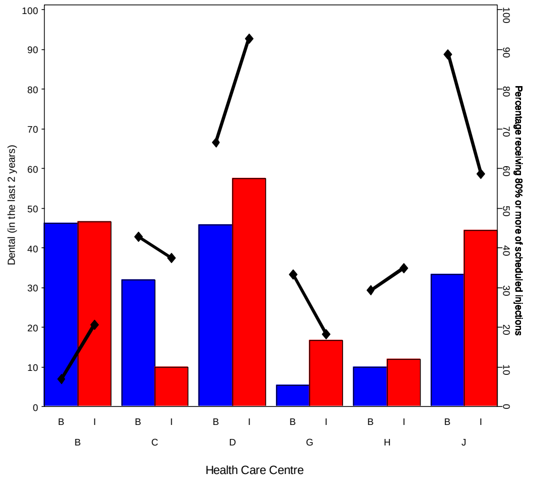

B. Documentation of dental review in the last 2 years

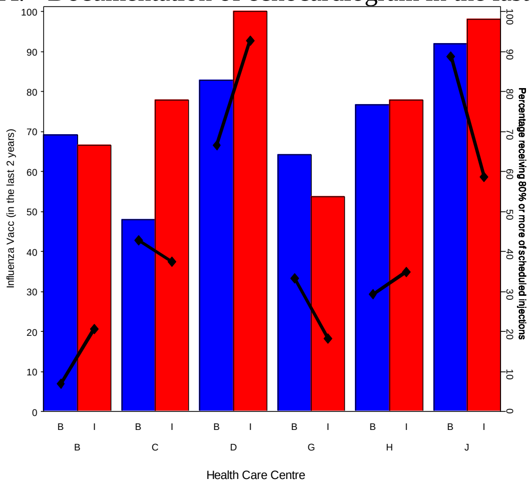

C. Documentation of receipt of influenza vaccine in the last 2 years

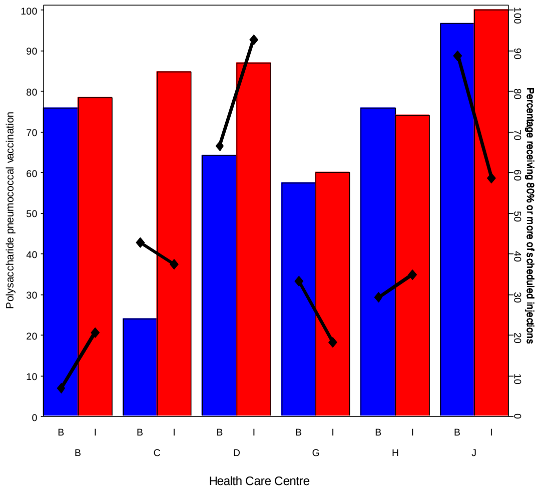

D. Documentation of receipt of adequate pneumococcal vaccine dosing

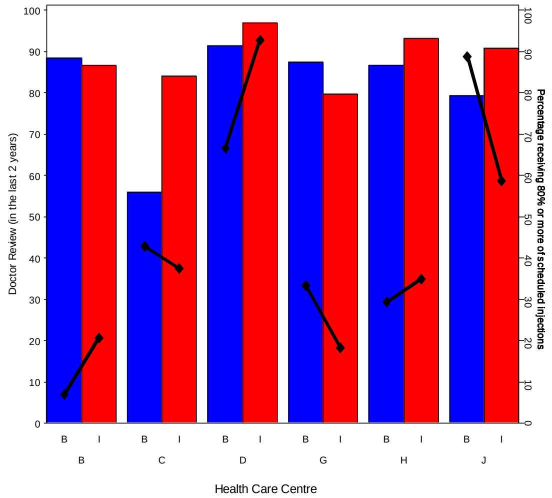

E. Documentation of any doctor review in the last 2 years

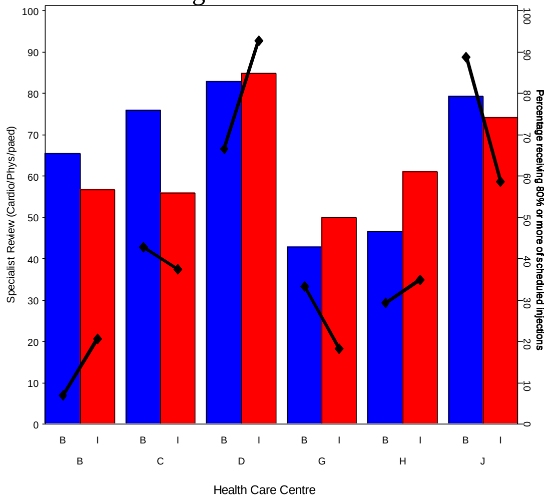

F. Documentation of specialist doctor review in the last 2 years

Colored bars show the percentage of audited individuals who had the variable of interest documented. Black dots and lines show adherence at that site during baseline and intensive phase. Five of the participating sites undertook audits during baseline and intensive phases.
